# Kidney Veno-Muscular Characteristics and Kidney Disease Progression: A Native Kidney-Biopsy Study

**DOI:** 10.1016/j.xkme.2023.100733

**Published:** 2023-10-05

**Authors:** Kenji Tsuji, Hiroyuki Nakanoh, Kensaku Takahashi, Takafumi Morita, Yizhen Sang, Kazuhiko Fukushima, Natsumi Matsuoka-Uchiyama, Yasuhiro Onishi, Haruhito A. Uchida, Shinji Kitamura, Jun Wada

**Affiliations:** 1 Department of Nephrology, Rheumatology, Endocrinology and Metabolism, Okayama University Faculty of Medicine, Dentistry and Pharmaceutical Sciences, Japan; 2 Department of Chronic Kidney Disease and Cardiovascular Disease, Okayama University Faculty of Medicine, Dentistry and Pharmaceutical Sciences, Japan

**Keywords:** Veno-muscular complex, kidney pathology, kidney biopsy, interstitial fibrosis/tubular atrophy, inflammation

## Abstract

**Rationale & Objective:**

Assessment of kidney biopsies provides crucial information for diagnosis and disease activity, as well as prognostic value. Kidney-biopsy specimens occasionally contain veno-muscular complex (VMC), which consists of muscle tissues around the kidney venous system in the corticomedullary region. However, the role of VMC and the clinical significance of VMC variants are poorly understood. In the present study, we investigated kidney prognostic values of VMC variants.

**Study Design:**

Retrospective cohort study.

**Setting & Participants:**

Among 808 patients who underwent a kidney biopsy from 2011 to 2019, 246 patients whose kidney biopsy specimens contained VMC were enrolled.

**Predictors:**

VMC variants; inflammatory-VMC (an infiltration of ≥80 inflammatory cells/mm^2^-VMC area) and VMC hypertrophy (hyper-VMC, a VMC average width ≥850 μm), and the interstitial fibrosis/tubular atrophy (IFTA) score.

**Outcomes:**

A decline in estimated glomerular filtration rate (eGFR) ≥40% from the baseline or commencement of kidney replacement therapy.

**Analytical Approach:**

Cox proportional hazards model.

**Results:**

Among 246 patients with data on VMC, mean baseline eGFR was 56.0±25.6 ml/min per 1.73 m^2^; 80 had high inflammatory-VMC, and 62 had VMC hypertrophy. There were 51 kidney events over median follow-up of 2.5 years. We analyzed 2 VMC variants. Multivariable logistic regression analysis revealed that eGFR negatively correlated with the presence of both inflammatory-VMC and hyper-VMC. A Cox proportional hazards analysis revealed that inflammatory-VMC (but not hyper-VMC) was independently associated with the primary outcome after adjustments for known risk factors of progression, including proteinuria, eGFR, and the interstitial fibrosis/tubular atrophy (IFTA) score (hazard ratio, 1.97; 95% confidence interval, 1.00-3.91).

**Limitations:**

Single-center study and small sample size.

**Conclusions:**

Assessment of inflammatory-VMC provides additional kidney prognostic information to known indicators of kidney disease progression in patients who undergo kidney biopsy.

**Plain-Language Summary:**

Assessment of kidney biopsies provides crucial information for diagnosis, disease activity, and prognostic value. Kidney-biopsy specimens occasionally contain veno-muscular complex (VMC), which consists of muscle tissues around the kidney venous system. Currently, the role of VMC in kidney health and diseases and the clinical significance of VMC variants are poorly understood. In the present study, we have shown that an infiltration of ≥80 inflammatory cells/mm^2^-VMC area (inflammatory-VMC) is independently associated with kidney disease progression after adjustments for known risk factors of progression. Therefore, assessment of inflammatory-VMC provides additional kidney prognostic information to known indicators of kidney disease progression in patients who undergo kidney biopsy.

## Graphical abstract

**Figure:**
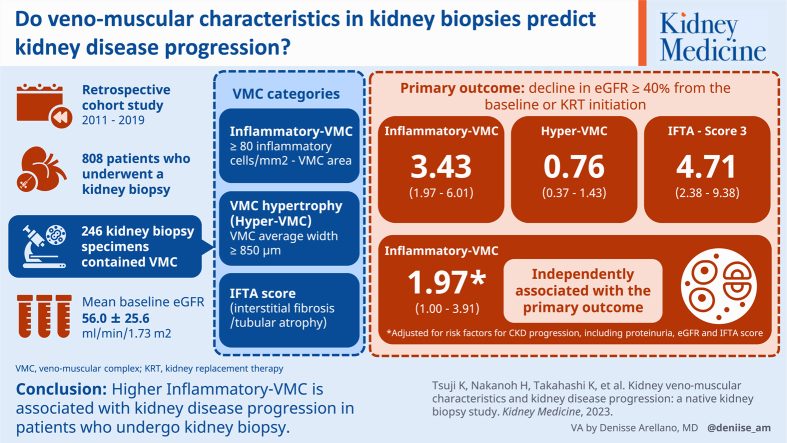

Proteinuria and serum creatinine (s-Cr) levels are widely recognized as predictors for kidney disease prognosis.^1^^,^^2^ In addition to these clinical parameters, histopathological assessments via kidney biopsy provide additional information regarding diagnosis, indication of disease activity, chronicity, and prognosis.^3^, ^4^, ^5^ A recent systemic analysis revealed good kidney prognostic value for the evaluation of histopathologic lesions, including interstitial fibrosis/tubular atrophy (IFTA), global glomerulosclerosis, and arterial sclerosis.^6^ Among these factors, IFTA is widely applied as a strong prognostic indicator of progression of kidney diseases.^7^ This prognostic information is critically important for the determination of patient treatments.

Kidney-biopsy specimens sometimes contain muscle tissues around the kidney venous system in the corticomedullary region, known as veno-muscular complex (VMC). It has been reported that VMC exists specifically in human kidneys^8^ and does not exist in other mammalian kidneys, such as rodent kidneys.^9^ VMC is located primarily around the arcuate artery, and consists with arcuate arteries, arcuate veins and the coiled artery.^10^ Although it has been suggested that VMC regulates the kidney circulation system,^8^ VMC’s role in kidney health and diseases and the clinical significance of VMC variants are poorly understood and there is currently no reported systemic analysis of VMC variants. Careful VMC evaluation in different kidney-biopsy specimens revealed several VMC variants, such as hypertrophy and inflammatory cell infiltration, in the VMC region. In the present study, we conducted a retrospective observational cohort study to investigate the clinical significance of VMC variants.

## Methods

### Study Design and Participants

**Figure 1:**
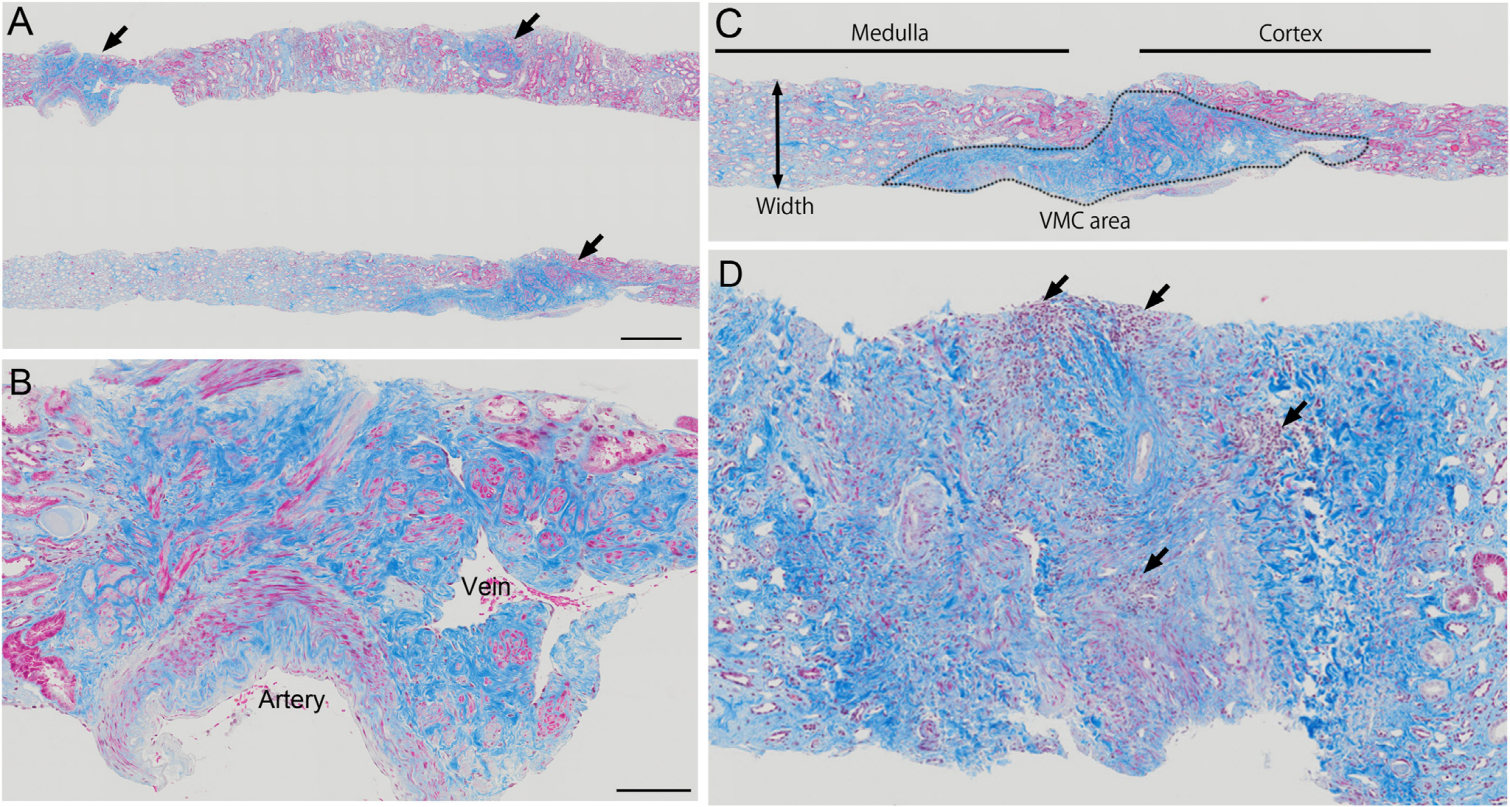
The variants of veno-muscular complex. (A, B) The image of light microscopic pictures in kidney tissue by Masson’s trichrome staining, showing the VMC (arrows) in the corticomedullary region. Scale bars: a, 500 μm; b, 100 μm. (C) A representative image of light microscopic pictures in the hyper-VMC-positive kidney tissue by Masson’s trichrome staining, showing the calculation of area/width of VMC (VMC-A/W) in the hyper-VMC-positive kidney tissue; VMC-A/W = area of VMC (μm^2^) / width (μm). (D) A representative image of light microscopic pictures in inflammatory-VMC-positive kidney tissue by Masson’s trichrome staining, showing the inflammatory cell infiltration (arrows) in VMC. VMC, veno-muscular complex.

We retrospectively reviewed 808 adult patients who underwent a native kidney biopsy in the Okayama University Hospital (Okayama, Japan) between January 2011 and December 2019, and we extracted 261 patients whose kidney biopsy specimens contained VMC (Figure 1 A and B). The exclusion criteria were estimated glomerular filtration rate (eGFR) ≤10 ml/min per 1.73 m^2^, patients receiving dialysis at the time of the kidney biopsy, or age <18 years. Consequently, 246 patients were enrolled in the study (Figure S1). The study protocol was approved by the ethics committee of Okayama University Hospital Institutional Review Board (accredited ISO9001/2000), Okayama, Japan (approval no. 2106-017), and is in accordance with the principles of the Declaration of Helsinki. No written informed consent was obtained because the study was considered exempt. Instead, the contents of our research were posted on our department’s homepage and in the hospital for public informed consent.

### Histological Parameters

Kidney tissues were obtained using either a 16- or 18-gauge needle, and specimens were processed for light microscopy, immunofluorescent staining, and electron microscopy. For light microscopy, specimens were stained with hematoxylin and eosin, periodic acid–Schiff, periodic acid–methenamine silver, and Masson’s trichrome, according to routine methods. All biopsies were adjudicated to determine histologic scores and diagnosis by at least 2 experienced nephrologists, through conference discussions based on patients’ medical histories, physical information, clinical examinations, and pathological findings. The IFTA score and total Inflammation score (Inflammation score) were graded on a scale of 0, 1, 2, and 3 (≤10%, 11%-25%, 26%-50%, and >50%, respectively) according to previous reports and the Banff classification of kidney allograft pathology.^6^^,^^7^^,^^11^

### Evaluation of Veno-Muscular Complex

VMC borders were clearly distinguishable using Masson’s trichrome staining because VMC contains connective tissue. VMC areas and widths were measured using virtual microscopy (OLYMPUS, Tokyo, Japan) to evaluate Masson’s trichrome–stained slides. Because either 16- or 18-gauge needle was used for kidney biopsy, the thickness of kidney specimens may vary between the samples, which may cause sampling bias. To adjust samples’ thicknesses for fair comparisons of VMC areas as possible, we applied the area/width of VMC (VMC-A/W) calculated as follows: VMC-A/W = area of VMC (μm^2^) /width (μm) of biopsied kidney tissue (Figure 1C), where VMC-A/W indicates the length of VMCs in kidney specimens obtained by adjusting this width.

### Clinical Parameters

The following clinicopathologic characteristics were collected at the time of kidney biopsies: age, sex, body mass index (BMI), hemoglobin A_1c_ (HbA_1c_), the use of angiotensin–converting enzyme inhibitor (ACEi) and/or angiotensin II receptor blocker (ARB), blood pressure, history of smoking, s-Cr and eGFR levels, urinary protein levels, and hematuria (>5 erythrocytes per high power field). eGFR was evaluated using the equation developed by the Japanese Society of Nephrology.^12^ Hypertension was defined as blood pressure (BP) ≥140/90 mm Hg at the time of the kidney biopsy or use of antihypertensive drugs. HbA_1c_ data are presented as National Glycohemoglobin Standardization Program values according to the recommendations of the Japanese Diabetes Society and the International Federation of Clinical Chemistry.^13^

### Exposure and Outcomes

The primary exposures were hypertrophy of VMC (hyper-VMC) and inflammatory cell infiltration in VMC (inflammatory-VMC), as well as known predictive measurements, including IFTA scores.^6^ The primary endpoint was defined as an eGFR decline of  ≥40% or the commencement of kidney replacement therapy (dialysis or kidney transplantation). eGFR levels were obtained from electronic medical records during follow-up.

### Statistical Analysis

Data were summarized as percentages for categorical variables and mean ± standard deviation (SD) or median (interquartile range [IQR]) for continuous variables. Skewed variables (proteinuria and VMC-A/W) underwent a natural logarithmic transformation before the analysis. Categorical variables were analyzed using a χ^2^ test, whereas continuous variables were compared using *t* test or a Mann-Whitney *U* test, as appropriate. *P* value for trend was calculated by the Cochran-Armitage trend test or the Jonckheere-Terpstra test for the distribution of each clinical parameter, stratified by IFTA score and Inflammation score. Correlations between VMC-A/W and eGFR were evaluated using Spearman correlation analysis. To evaluate the association of variates with hyper-VMC or inflammatory-VMC, we performed univariate and multivariable logistic regression analyses, in which we evaluated the prevalence odds ratio (OR) for hyper-VMC or inflammatory-VMC. We used multiple linear regression analysis to evaluate the association of variates with a dependent variable; VMC-A/W were calculated as continuous variables. For these analyses, candidate variables included age, male gender, eGFR, diabetes mellitus, hypertension, BMI, urinary protein levels, ACEi or ARB intake, and hematuria. Factors with a strong confounding influence on each other were not included in the same model. We applied the Kaplan-Meier method to estimate cumulative kidney survival for hyper-VMC, inflammatory-VMC, IFTA score, and Inflammation score; kidney survival rates were evaluated using the log-rank test. We censored patients who did not reach the endpoint by the 10-year follow-up date or the last recorded visit on October 31, 2021. We identified the number of patients at risk of reaching the endpoint from the date of their kidney biopsy. The Cox proportional hazards model was applied to calculate the hazard ratio (HR) and 95% confidence interval (95% CI) for the interval-censored times to event. All the Cox models were first applied without adjustments and then adjusted with a multivariable for covariates that were selected as potential confounders based on biological plausibility and previous reports.^6^^,^^7^ Statistical analyses were performed by JMP version 9.0.0 or 17.0.0 (SAS Institute, Inc, Cary, NC) and STATA V15.0 (StataCorp, College Station, TX, USA). All *P* values were calculated as two-sided. Significance was defined as *P* <0.05.

## Results

### Patient Characteristics at the Time of Kidney Biopsy

**Figure 2:**
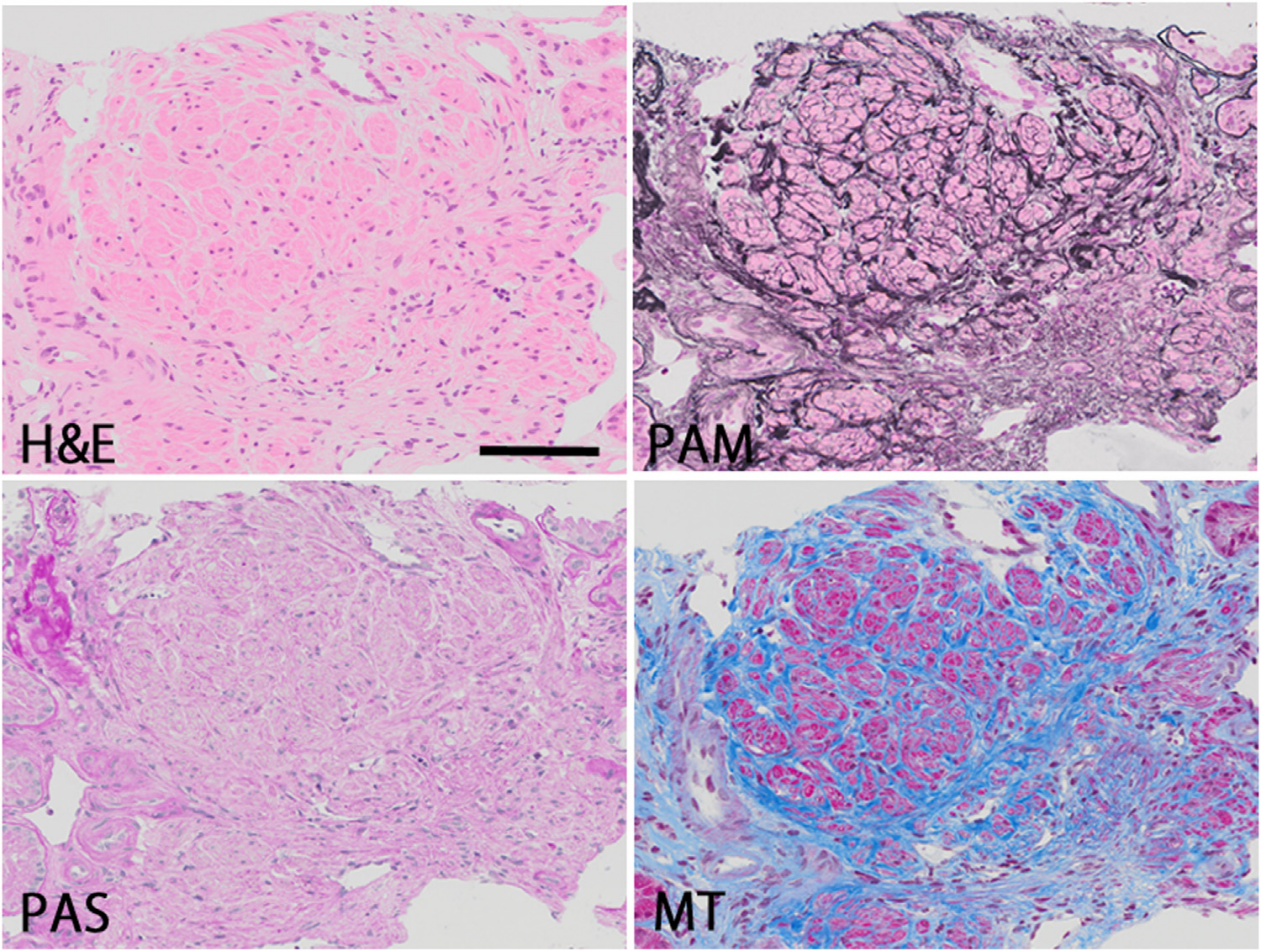
Images for veno-muscular complex. Representative images of light microscopic pictures for VMC. VMC, veno-muscular complex; H&E, Hematoxylin and Eosin; PAM, Periodic acid–methenamine silver; PAS, *Periodic acid–Schiff*; MT, Masson’s trichrome staining. Scale bar: 100 μm.

Among 808 patients who underwent a kidney biopsy, kidney-biopsy specimens of 261 patients (32%) contained VMC region (Figure 2); 246 patients met the selection criteria and were enrolled in the analysis (Figure S1). Patient characteristics at the time of kidney biopsy and primary clinicopathologic diagnoses are presented in Tables S1 and S2, respectively. A mean age was 55.3±16.6 (SD) years; 131 patients (53%) were male, 53 patients (22%) had a history of diabetes mellitus, 141 patients (57%) had a history of hypertension, the mean eGFR was 56.0±25.6 (SD) ml/min per 1.73 m^2^, the median proteinuria was 1.04 g/gCr (IQR, 0.37-3.84) and the median VMC-A/W was 541 μm (IQR, 347-814). To consider the definition of inflammatory-VMC, we counted the number of inflammatory cells in VMC region (Figure S2A), which revealed that 60% of the VMC regions did not have inflammatory cell infiltration, while 29% of the VMC regions had inflammatory cells ≥200/mm^2^-VMC area. Based on Table S3 results of univariate and multivariable Cox proportional hazard models, we defined inflammatory-VMC as infiltration of ≥80 inflammatory cells/mm^2^-VMC area (Figure 1D). A total of 80 patients (33%) were inflammatory-VMC-positive. Unlike the results of inflammatory-VMC, univariate and multivariable Cox proportional hazard models for the hypertrophy of VMC did not show significant results (Table S4). Considering the first quartile of 814 μm, we defined hyper-VMC as ≥850 μm VMC-A/W (Figure 1C), where 62 patients (25%) were hyper-VMC-positive. Clinical and histopathological variables, stratified by IFTA score, Inflammation score, inflammatory-VMC, and hyper-VMC, were as follows (Table 1, Table 2, Table S5, and Table S6): patients with inflammatory-VMC had higher s-Cr levels, IFTA scores, Inflammation scores and VMC-A/W, more frequent hypertension, took more ACE-I/ARB and statin, and had lower eGFR levels and BMI. Patients with hyper-VMC had lower eGFR levels.

**Table 1:** Clinical and Histopathological Findings for All Patients and for Patients Stratified by the Inflammatory-VMC

Inflammatory-VMC	All patients (N=246)	Inflammatory-VMC (-) (n=166)	Inflammatory-VMC (+) (n=80)	*P* value
Men	131 (53%)	89 (54%)	42 (52%)	0.87
Age, y	55.3±16.6	54.7±16.6	56.4±16.4	0.45
BMI, kg/m^2^	22.7±3.5	23.1±3.4	22.0±3.8	0.02^a^
sBP, mm Hg	128.5±18.6	127.5±17.3	130.5±21.6	0.23
dBP, mm Hg	79.1±13.2	78.8±12.2	79.9±15.1	0.56
sCr, mg/dl	1.26±0.84	1.15±0.81	1.48±0.87	0.005^b^
eGFR, mi/min/1.73 m^2^	56.0±25.6	61.4±26.5	44.9±19.3	< 0.001^b^
U-P, g/gCr	1.04 (IQR, 0.37-3.84)	0.95 (IQR, 0.32-3.19)	1.30 (IQR, 0.51-4.55)	0.04^a^
H**b**A_1c_	5.8±0.7 (%)	5.9±0.8 (%)	5.8±0.6 (%)	0.38
Hypertension	141 (57%)	86 (52%)	55 (68%)	0.01^a^
Diabetes mellitus	53 (22%)	37 (22%)	16 (20%)	0.68
ACE-I or ARB	102 (41%)	60 (36%)	42 (52%)	0.01^a^
Ca blockade	105 (43%)	65 (39%)	40 (49%)	0.11
Statin	65 (26%)	38 (23%)	27 (33%)	0.07
Hematuria	143 (58%)	96 (58%)	47 (58%)	0.94
IFTA score				
0	31 (13%)	30 (18%)	1 (1%)	< 0.001^b^
1	102 (41%)	83 (50%)	19 (24%)	
2	82 (33%)	45 (27%)	37 (46%)	
3	31 (13%)	9 (5%)	22 (27%)	
Inflammation score				
0	112 (46%)	93 (56%)	19 (24%)	< 0.001^b^
1	75 (30%)	52 (31%)	23 (29%)	
2	41 (17%)	16 (10%)	25 (31%)	
3	18 (7%)	5 (3%)	13 (16%)	
VMC-A/W, μm	541 (IQR, 347-814)	518 (IQR, 335-782)	614 (IQR, 392-875)	0.07

*Note*: Values reported as n (%) for categorical variables and mean ± SD or median (IQR) for continuous variables.

Abbreviation: VMC, veno-muscular complex; BMI, body mass index; sBP, systolic blood pressure; dBP, diastolic blood pressure; U-P, urinary protein excretion; sCr, serum creatinine; eGFR, estimated glomerular filtration rate; ACE-I, angiotensin–converting enzyme inhibitor; ARB, angiotensin II type 1 receptor blocker; IFTA, interstitial fibrosis and tubular atrophy; VMC-A/W, VMC average width; IQR, interquartile range; SD, standard deviation; HbA_1c_, Hemoglobin A_1c_.

a *P* < 0.05.

b *P* < 0.01.

**Table 2:** Clinical and Histopathological Findings for All Patients and for Patients Stratified by the Hyper-VMC

Hyper-VMC	All patients (N=246)	Hyper-VMC (-) (n=184)	Hyper-VMC (+) (n=62)	*P* value
Men	131 (53%)	97 (53%)	34 (55%)	0.77
Age, y	55.3±16.6	55.1±17.2	55.7±14.5	0.80
BMI, kg/m^2^	22.7±3.5	22.5±3.6	23.4±3.3	0.05
sBP, mm Hg	128.5±18.6	128.5±18.7	128.4±19.3	0.99
dBP, mm Hg	79.1±13.2	79.4±13.6	78.4±12.0	0.63
sCr, mg/dl	1.26±0.84	1.24±0.89	1.32±0.7	0.50
eGFR, mi/min/1.73 m^2^	56.0±25.6	57.9±26.2	50.2±22.8	0.04^a^
U-P,^a^ g/gCr	1.04 (IQR, 0.37-3.84)	1.06 (IQR, 0.37-4.25)	0.86 (IQR, 0.37-2.58)	0.29
HbA_**1c**_	5.8±0.7	5.8±0.7	5.8±0.7	0.46
Hypertension	141 (57%)	105 (57%)	36 (58%)	0.89
DM	53 (22%)	38 (21%)	15 (24%)	0.56
ACE-I or ARB	102 (41%)	75 (41%)	27 (44%)	0.70
Ca blockade	105 (43%)	76 (41%)	29 (47%)	0.45
Statin	65 (26%)	45 (25%)	20 (32%)	0.23
Hematuria	143 (58%)	109 (59%)	34 (55%)	0.63
IFTA score				
0	31 (13%)	23 (13%)	8 (13%)	0.24
1	102 (41%)	81 (44%)	21 (34%)	
2	82 (33%)	61 (33%)	21 (34%)	
3	31 (13%)	19 (10%)	12 (19%)	
Inflammation score				
0	112 (46%)	87 (47%)	25 (40%)	0.80
1	75 (30%)	55 (30%)	20 (32%)	
2	41 (17%)	29 (16%)	12 (19%)	
3	18 (7%)	13 (7%)	5 (8%)	
VMC-A/W,^a^ μm	541 (IQR, 347-814)	479 (IQR, 305-601)	1001 (IQR, 898-1398)	< 0.001^b^
Inflammatory-VMC	80 (335%)	56 (30%)	24 (39%)	0.23

*Note:* Values reported as n (%) for categorical variables and mean ± SD or median (IQR) for continuous variables.

Abbreviations: VMC, veno-muscular complex; BMI, body mass index; sBP, systolic blood pressure; dBP, diastolic blood pressure; sCr, serum creatinine; eGFR, estimated glomerular filtration rate; U-P, urinary protein excretion; HbA_1c_, Hemoglobin A_1c_; DM, Diabetes mellitus; ACE-I, angiotensin–converting enzyme inhibitor; ARB, angiotensin II type 1 receptor blocker; IFTA, interstitial fibrosis and tubular atrophy; VMC-A/W, VMC average width; IQR, interquartile range; SD, standard deviation.

a *P* < 0.05.

b *P* < 0.01.

### Predictors of VMC Variants

To explore the predictors of VMC variants, we first categorized patients as per the etiology of their kidney biopsies. Positive inflammatory-VMC rates were as follows: 25%, IgA nephropathy; 75%, acute tubulointerstitial nephritis (ATIN); 27%, focal segmental glomerulosclerosis; 7%, minimal change disease; 10%, membranous nephropathy; 42%, anti-neutrophil cytoplasmic antibody- related; 42%, diabetic nephropathy; 75%, lupus nephritis; 33%, minor glomerular abnormality (MGA); and 0%, thin basement membrane disease. Regarding VMC size, VMC-A/W of patients with ATIN were significantly larger than those of patients with MGA (Figure S2). These results indicate that prevalence of VMC variants may vary between etiologies. Next, we evaluated predictors of VMC variants using univariate and multivariable logistic regression analyses (Table 3 and 4). Factors independently associated with inflammatory-VMC were eGFR (OR, 0.73; 95% CI, 0.63-0.84, increased by 10ml/min per 1.73 m^2^) and BMI (OR, 0.89; 95% CI, 0.81-0.97), while factors independently associated with hyper-VMC were eGFR (OR, 0.85; 95% CI, 0.73-0.97, increased by 10 ml/min per 1.73 m^2^) and BMI (OR, 1.11; 95% CI, 1.01-1.22). To further analyze explanatory variables for VMC-A/W, we applied multiple linear regression analysis (Table S7), which revealed that eGFR levels and proteinuria were significantly associated with VMC-A/W (eGFR: unstandardized regression coefficient (B), -32.9; 95% CI: -53.9 to -11.8; U-P: (B), -13.7; 95% CI: -27.0 to -0.48). Relationships among the log-transformed VMC-A/W and clinical variables (Table S8) indicate that there were negative correlations between log-transformed VMC-A/W and eGFR.

**Table 3:** Univariate Logistic Regression Analysis for the Presence of VMC Variants and Clinical Variables

Variables	Inflammatory -VMC			Hyper-VMC		
	Odds ratio	95% CI	*P* value	Odds ratio	95% CI	*P* value
Age (increased by 5 y)	1.03	0.95-1.12	0.57	1.01	0.93-1.10	0.80
Gender	0.96	0.56 -1.63	0.87	1.09	0.61-1.95	0.77
eGFR (increased by 10 mL/min/1.73 m^2^)	0.74	0.65-0.84	<0.001^a^	0.88	0.78-0.99	0.04^b^
DM	0.85	0.43-1.62	0.63	1.23	0.61-2.39	0.56
Hypertension	2.11	1.21 -3.75	0.009^a^	1.04	0.58-1.88	0.89
BMI	0.91	0.84 -0.98	0.02^b^	1.08	0.99-1.17	0.06
U-P (increased by 1 g/gCr)	1.05	0.98-1.13	0.17	0.92	0.83-1.01	0.07
ACEi or ARB	2.03	1.19-3.51	0.01^b^	1.12	0.62-2.00	0.70
Hematuria	1.02	0.59-1.77	0.84	0.87	0.48-1.56	0.63
VMC-A/W	1.00	0.99-1.001	0.05	-	-	-

Abbreviations: eGFR, estimated glomerular filtration rate; DM, Diabetes mellitus; BMI, body mass index; U-P, urinary protein excretion; ACEi, angiotensin–converting enzyme inhibitor; ARB, angiotensin II type 1 receptor blocker; CI, confidence interval; VMC-A/W, veno-muscular complex average width.

a *P* < 0.01.

b *P* < 0.05.

**Table 4:** Multivariable Logistic Regression Analysis for the Presence of VMC Variants and Clinical Variables

Variables	Inflammatory-VMC			Hyper-VMC		
	Odds ratio	95% CI	*P* value	Odds ratio	95% CI	*P* value
Age (increased by 5 y)	0.93	0.83-1.04	0.21	0.96	0.86-1.07	0.46
Gender	0.95	0.50-1.79	0.87	0.89	0.47-1.69	0.72
eGFR (increased by 10 mL/min/1.73m^2^)	0.73	0.63-0.84	<0.001^a^	0.85	0.73-0.97	0.02^b^
DM	0.69	0.31-1.47	0.34	0.97	0.44-2.08	0.94
Hypertension	1.50	0.68-3.33	0.32	0.86	0.38-1.92	0.71
BMI	0.89	0.81-0.97	0.01^b^	1.11	1.01-1.22	0.03^b^
U-P (increased by 1 g/gCr)	1.06	0.97-1.16	0.17	0.91	0.82-1.00	0.05
ACEi or ARB	1.64	0.80-3.45	0.18	1.10	0.51-2.40	0.80
Hematuria	1.01	0.543-1.90	0.97	0.87	0.47-1.64	0.67
VMC-A/W	1.00	0.99-1.001	0.12	-	-	-

Abbreviations: eGFR, estimated glomerular filtration rate; DM, Diabetes mellitus; BMI, body mass index; U-P, urinary protein excretion; ACEi, angiotensin–converting enzyme inhibitor; ARB, angiotensin II type 1 receptor blocker; CI, confidence interval; VMC-A/W, veno-muscular complex average width.

a *P* < 0.01.

b *P* < 0.05.

### Prognostic Significance of Histopathologic Lesions

**Figure 3:**
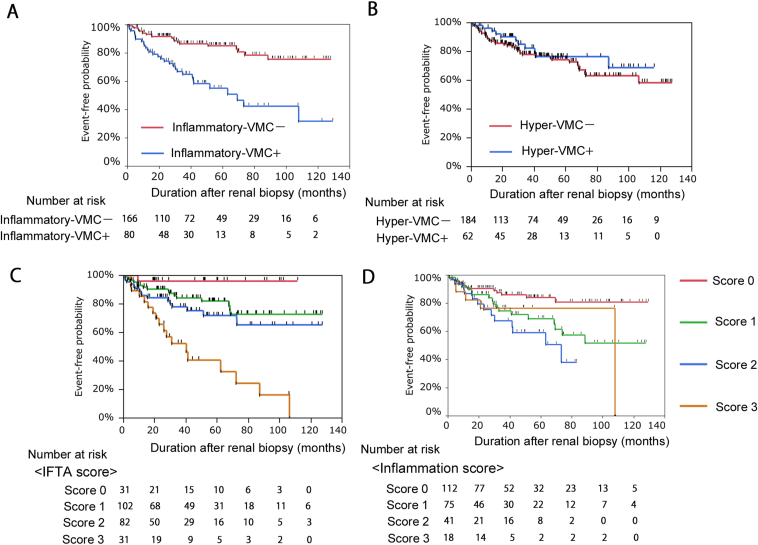
Kidney survival rate stratified by hyper-VMC, inflammatory-VMC, IFTA score and Inflammation score. (A) Kidney survival rate stratified by inflammatory-VMC. Log-rank test: *P*<0.001. (B) Kidney survival rate stratified by Hyper-VMC. Log-rank test: *P*=0.422. (B) Kidney survival rate stratified by IFTA score. Log-rank test: *P*<0.001. (D) Kidney survival rate stratified by Inflammation score. Log-rank test: *P*=0.008. VMC, veno-muscular complex.

During the median follow-up period of 2.5 years (IQR, 0.7-5.0), 51 patients experienced a kidney outcome of  ≥40% eGFR decline or started kidney replacement therapy. Kaplan-Meier curves for kidney survival stratified by inflammatory-VMC, hyper-VMC, IFTA score, and Inflammation score (Figure 3) indicate that inflammatory-VMC and higher IFTA scores and Inflammation scores were significantly associated with an increased risk of kidney outcome; however, there was no significant association of kidney survival with hyper-VMC. The adjusted HRs of inflammatory-VMC, IFTA score, and Inflammation score for kidney endpoint were calculated (Table 5). A Cox proportional hazards analysis revealed that inflammatory-VMC and IFTA scores were positively associated with kidney endpoint after adjustments with eGFR and proteinuria, whereas the association of Inflammation scores with the outcome was attenuated. In addition, the association of inflammatory-VMC with kidney endpoint remained positive after further adjustment using the IFTA score (HR 1.97; 95% CI, 1.003-3.91). Despite the prognostic value of inflammatory-VMC, correction by VMC area is relatively complicated for routine assessments of kidney biopsy. Therefore, we also examined values of inflammatory-VMC without corrections to simplify the inflammatory-VMC definition. Patient numbers stratified by inflammatory cells with or without area corrections (Figure S2) indicated a strong match between infiltration of ≥80 inflammatory cells/mm^2^-VMC area (inflammatory-VMC) and infiltration of ≥20 inflammatory cells/VMC (inflammatory-VMC without area correction). In addition, analyses of Kaplan-Meier curves (Figure S4) and a Cox proportional hazards (Table S9) for kidney survival revealed that an infiltration of ≥20 inflammatory cells/VMC was significantly associated with an increased risk of kidney outcome after adjustments with eGFR and proteinuria (HR 2.62; 95% CI, 1.44-4.89). Collectively, an infiltration of ≥20 inflammatory cells/VMC could be an alternative definition for inflammatory-VMC under routine assessments of kidney biopsy.

**Table 5:** Univariate and Multivariable Cox Proportional Hazard Models for Inflammatory-VMC, Hyper-VMC, IFTA Score, and Inflammation Score

	Univariate HR [95% CI]	Model 1 HR [95% CI]	Model 2 HR [95% CI]	Model 3 HR [95% CI]
Inflammatory-VMC				
Absent	Reference	Reference	Reference	Reference
Present	3.43 (1.97-6.01)	3.39 (1.94-6.01)	2.69 (1.46-5.03)	1.97 (1.00-3.91)
Hyper-VMC				
Absent	Reference	Reference	Reference	Reference
Present	0.76 (0.37-1.43)	0.74 (0.36-1.40)	0.82 (0.40-1.58)	0.78 (0.37-1.50)
IFTA score				
Score 0	0.21 (0.03-1.79)	0.24 (0.03-1.79)		
Score 1	Reference	Reference		
Score 2	1.52 (0.75-3.06)	1.48 (0.73-2.99)		
Score 3	4.71 (2.38-9.38)	4.80 (2.43-9.60)		
Per score	2.30 (1.66-3.20)	2.29 (1.65-3.22)	2.05 (1.35-3.16)	-
Inflammation score				
Score 0	0.44 (0.22-0.87)	0.42 (0.20-0.84)		
Score 1	Reference	Reference		
Score 2	1.51 (0.73-3.06)	1.37 (0.65-2.80)		
Score 3	1.08 (0.40-2.90)	1.07 (0.40-2.90)		
Per score	1.47 (1.13-1.90)	1.47 (1.13-1.91)	1.11 (0.81-1.51)	0.85 (0.601.22)

*Note:* Model 1, adjusted for age and sex. Model 2, adjusted for urinary protein excretion and eGFR. Model 3, adjusted for the covariates in model 2 and IFTA.

Abbreviations: VMC, veno-muscular complex; IFTA, interstitial fibrosis and tubular atrophy; HR, hazard ratio; CI, confidence interval.

## Discussion

In this cohort study, we found that inflammatory-VMC is an independent predictor for kidney disease progression. To our knowledge, this is the first study that has analyzed VMC variants systemically using kidney-biopsy specimens. The kidney is a vascular and encapsulated organ that is sensitive to insufficient or excess blood flow^14^, as well as venous congestion. Normally, an autoregulation in-out balance system regulates input from arteries and output into venous flow. There are reports that increased central venous pressure is associated with impaired kidney function via kidney congestion.^15^ Indeed, the relevance of congestion in cardio-renal syndrome^16^ and decreased urine flow through increased venous pressure in canine kidneys^17^ has been reported. That elevation of central venous pressure is the strongest hemodynamic determinant for worsening kidney function has also been reported, highlighting the importance of venous congestion.^18^ Regarding kidney venous return control, Barrie et al^19^ identified the existence of coiled renal arteries functioning as an arteriovenous (AV) shunt, indicated by the arcuate sponge.^19^ In addition, VMC has been reported as a contributor to venous regulation.

**Figure 4:**
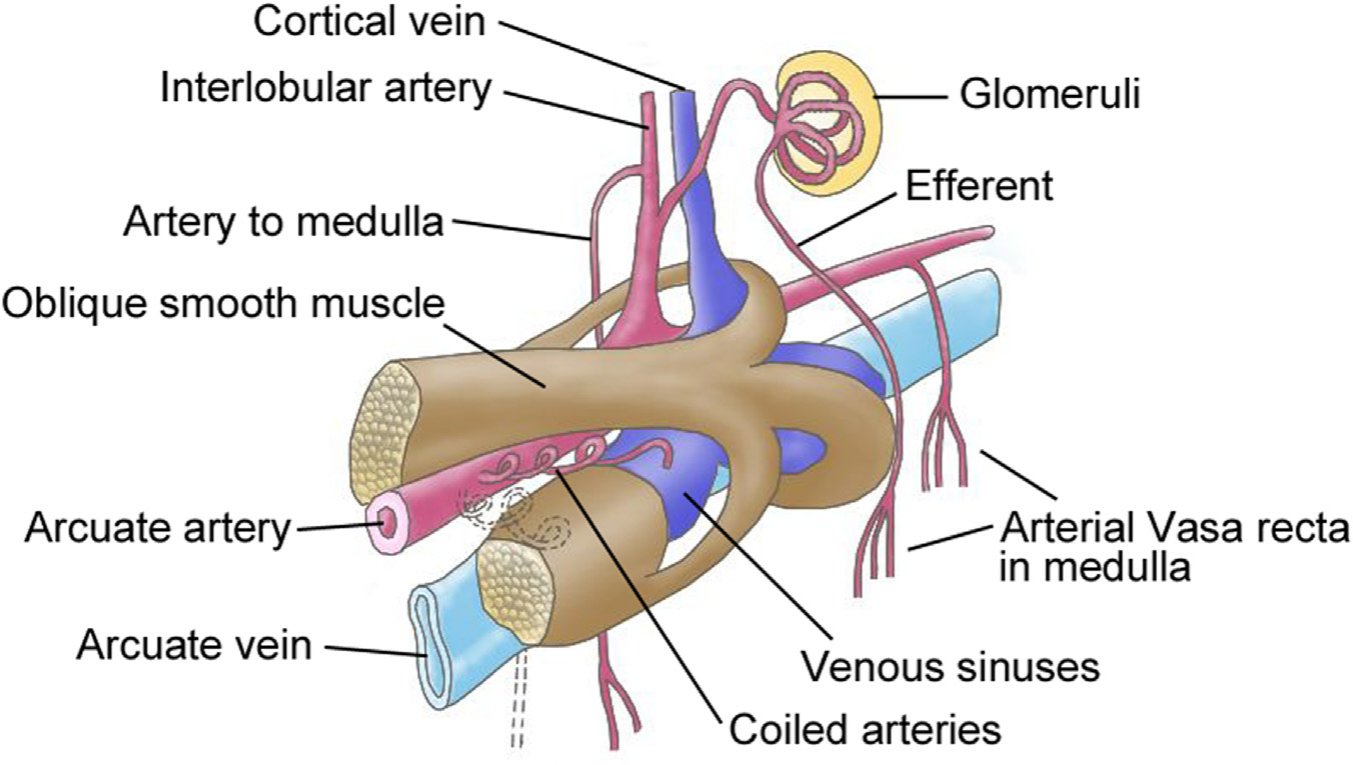
Scheme of veno-muscular complex.

Currently, there are only few reports indicating kidney VMC. Onuki et al^9^ reported the existence of VMC in the kidney corticomedullary region in 1976.^9^ They indicated that VMC, which is primarily located around the arcuate artery, consists of peri-arterial smooth muscle bundle, veno-muscular smooth muscle, free smooth muscle, coiled arteries, large venous sinuses, the trunk of the vasa recta, and the lymphatics; Figure 4 shows such a VMC scheme. Similarly, Takeuchi et al^10^ indicated that VMC is primarily composed of smooth muscle bundles in the kidney corticomedullary region, involves arcuate arteries and arcuate veins, and that the coiled artery and nerve bundles are intertwined; therefore, VMC may regulate venous blood flow and venous pressure through venous contraction.^10^ Matsuo et al^20^ further explored VMC’s origin using continuous sections and revealed that VMC’s smooth muscle is continuous, with the kidney calyx’s smooth muscle and the smooth muscle group surrounding the arched and interlobar veins.^20^ The smooth muscle coiled around the kidney calyx squeeze urine from the kidney papilla via muscle contractions (known as the milking effect);^21^ Matsuo et al^20^ suggested that VMC might drain not only urine but also venous blood out of the kidney.^20^ In fact, after observing the systolic and diastolic histology of the kidney calyx muscle,^22^ they noted that there were no red blood cells in the kidney papilla interstitium’s blood vessels during muscle systole, whereas red blood cells filled blood vessels during muscle diastole, which supported their hypothesis. Collectively, VMC may have a crucial role in regulating kidney blood flow through muscle contraction.

What happens during VMC disorder? Matsuo et al^8^ suggested that abnormality of VMC might impair kidney circulation, which results in a decrease in GFR.^8^ In fact, it has been suggested that the VMC fibrosis seen in liver cirrhosis is associated with hepatorenal syndrome, which significantly involves kidney circulation.^8^ Subsequently, Furuno et al reported an autopsy case in which hydrocele, bleeding and the extension of smooth muscle in the VMC were observed.^23^ Similarly, hypertrophy or inflammatory cell infiltration in VMC may cause dysfunction through a kidney venous flow defect. Increased interstitial fibrosis and inflammation have been shown to predict disease progression in IgA nephropathy and lupus nephritis.^24^^,^^25^ Accumulation of interstitial inflammatory cells promotes interstitial fibrosis by enhancing antigen presentation and autoantibody production.^26^^,^^27^ VMC’s physiological function might also be impaired by inflammation, leading to further abnormal kidney function. Importantly, we found that inflammatory-VMC could predict kidney disease progression. While patients with inflammatory-VMC had higher IFTA scores and Inflammation scores, inflammatory-VMC was independently associated with the kidney disease progression after adjustments for IFTA score unlike Inflammation scores, suggesting that inflammatory-VMC may not be just a marker of inflammation in the tubulointerstitium, and may be involved in kidney disease progression. Further study would be required to uncover VMC’s function and disorder, inflammatory-VMC.

This study had several limitations. First, there might have been selection bias because of the relatively small sample size in a single university hospital as well as the variety of etiologies. Further analysis with large number of samples from several departments would be necessary to support our conclusion. Second, VMC is not always obtained from kidney biopsies, partly because biopsy samples do not necessarily contain corticomedullary regions where VMC normally exist. In addition, it has been reported that 2 or 3 VMC regions are usually found in the sagittal section of a human kidney specimen, indicating that corticomedullary regions do not necessarily contain VMC. Because 32% (261 out of 808 patients) of biopsy specimens contained VMC in this study, it was difficult to evaluate VMC variants in the remaining 68% of the cases. Third, the possibility of false negatives for both hyper-VMC and inflammatory-VMC could not be excluded because of the limited kidney-biopsy sample size; this would have affected our results. In particular, a fair calculation of VMC area from a kidney biopsy is relatively difficult. Although we made the correction by VMC width to quantify VMC area as fairly as possible, we likely had significant bias regarding VMC size. Therefore, the absence of hyper-VMC’s prognostic value for kidney outcome in this study does not necessarily imply that VMC hypertrophy does not affect kidney function. Future large cross-sectional studies that analyze normal anatomy in nephrectomy specimens are required to consider and overcome the VMC size problem. Despite these limitations, we systemically analyzed VMC size for the first time and revealed the association between VMC variants and several clinical variables, as well as the prognostic value of inflammatory-VMC for kidney outcome, and suggested the possible involvement of pathophysiology under some kidney disease conditions.

In conclusion, the assessment of inflammatory-VMC adds prognostic value to known indicators of kidney disease progression in patients who undergo kidney biopsies. Accordingly, it seems important to evaluate kidney VMC inflammatory cell infiltration in patients with VMC regions in their kidney biopsy specimens. Further analysis is required to determine the clinical significance of VMC hypertrophy, as well as VMC roles.
