# Supplementary material for: Kidney Veno-Muscular Characteristics and Kidney Disease Progression: A Native Kidney-Biopsy Study
Source: Kidney Med. 2023 Oct 5;5(12):100733. doi: 10.1016/j.xkme.2023.100733 (PMC10692955; doi:10.1016/j.xkme.2023.100733)
Supplement: Supplementary File 1 (PDF) — Figure S1-S4. Table S1-S9. [file mmc1.pdf]

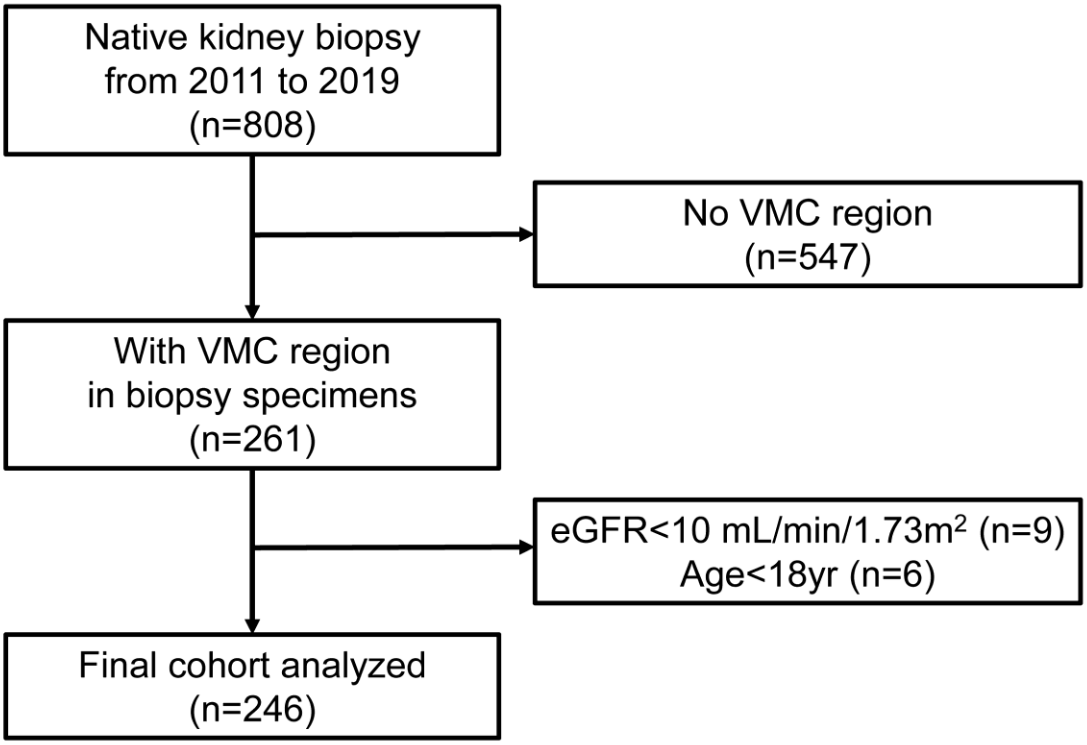

Figure S1. Flowchart of study participants.

VMC, veno-muscular complex.

A

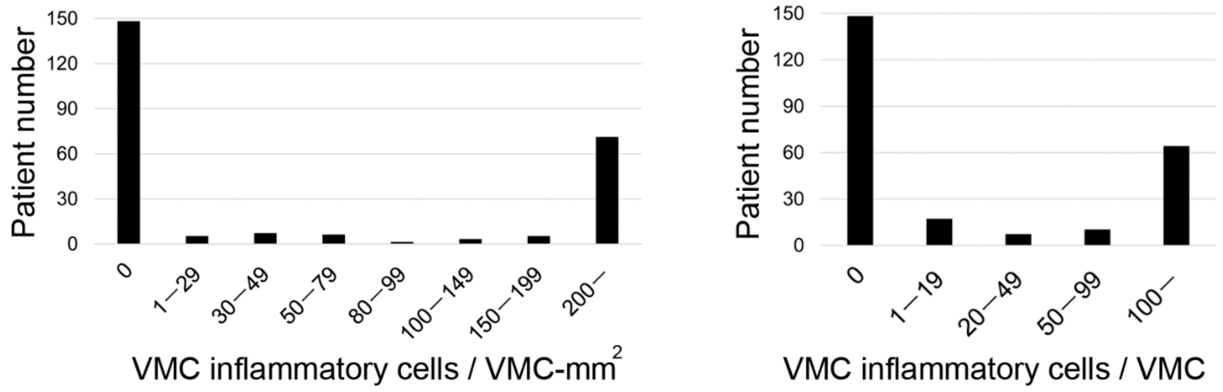

B

		VMC inflammatory cells / VMC-mm²	
		0-79	80-
VMC inflammatory cells / VMC	0-19	165	0
	20-	1	80

Figure S2. Stratification by VMC inflammatory cells/VMC-mm^2 or inflammatory cells/VMC

(A) Patient number in VMC inflammatory cells/VMC-mm^2 and VMC inflammatory cells/VMC. (B) Patient number stratified by VMC inflammatory cells/VMC-mm^2 and VMC inflammatory cells/VMC. VMC, veno-muscular complex.

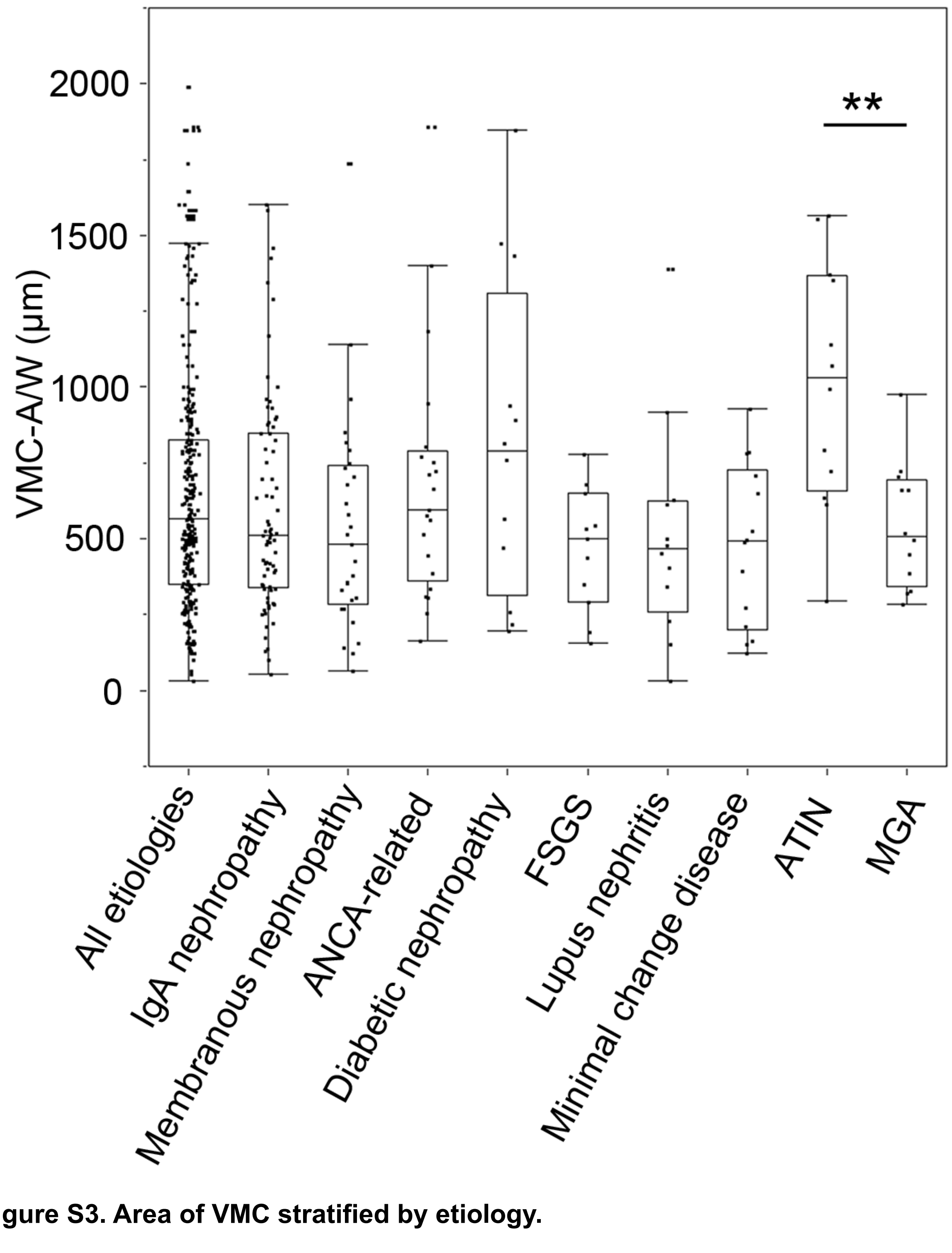

Figure S3. Area of VMC stratified by etiology.

VMC-A/W, area/width of veno-muscular complex; ANCA, anti-neutrophil cytoplasmic antibody; ATIN, acute tubulointerstitial nephritis; FSGS, focal segmental glomerulonephritis; MGA, minor glomerular abnormality.

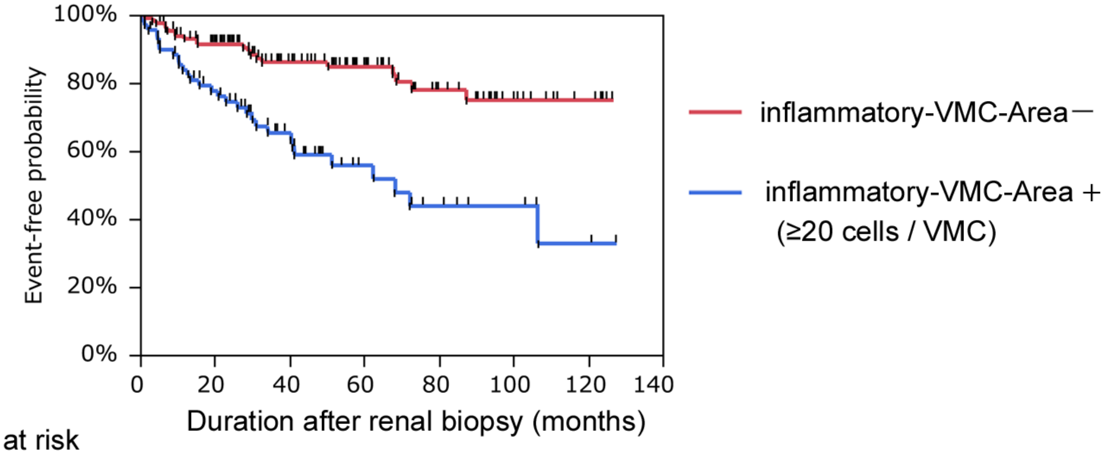

Number at risk

inflammatory-VMC-Area－	165	109	71	48	28	15	6
inflammatory-VMC-Area＋	81	49	31	14	9	6	3

Figure S4. Kidney survival rate stratified by Inflammatory-VMC-Area.

Kidney survival rate stratified by Inflammatory-VMC-Area. Log rank test: p<0.001. VMC, veno-muscular complex.

Table S1. Clinical and histopathological characteristics for all patients.

Characteristics	n=246
Men, n (%)	131 (53)
Age, yr.	55.3±16.6
BMI, kg/m^2	22.7±3.5
sBP, mmHg	128.5±18.6
dBP, mmHg	79.1±13.2
sCr, mg/dl	1.26±0.84
eGFR, ml/min/1.73 m^2	56.0±25.6
U-P,[a] g/gCr	1.04 (0.37-3.84)
HbA1c, n (%)	5.8±0.7
Hypertension, n (%)	141 (57)
DM, n (%)	53 (22)
ACE-I or ARB, n (%)	102 (41)
Ca blockade, n (%)	105 (43)
Statin, n (%)	65 (26)
Hematuria, n (%)	143 (58)
IFTA score, n (%)	
0	31 (13)
1	102 (41)
2	82 (33)
3	31 (13)
Inflammation score, n (%)	
0	112 (46)
1	75 (30)
2	41 (17)
3	18 (7)
VMC A/W, [a] µm	541 (347-814)
0-150 µm, n (%)	10 (4)
151-350, n (%)	53 (22)
351-550, n (%)	62 (25)
551-850, n (%)	67 (27)
851-1200, n (%)	32 (13)
1201-1500, n (%)	12 (5)
1501-, n (%)	10 (4)
Inflammatory-VMC, n (%)	80 (33)

BMI, body mass index; sBP, systolic BP; dBP, diastolic BP; sCr, serum creatinine; eGFR, estimated glomerular filtration rate; HbA1c, Hemoglobin A1c; DM, Diabetes mellitus; U-P, urinary protein excretion; ACE-I, angiotensin–converting enzyme inhibitor; ARB, angiotensin II type 1 receptor blocker; IFTA, interstitial fibrosis and tubular atrophy; VMC, veno-muscular complex; VMC-A/W, VMC average width. [a] Median (interquartile range).

Table S2. Primary clinicopathologic diagnoses.

Glomerulonephritis	Genetic	Metabolic	Tubulointerstitial	Others
IgA nephropathy (77)	Alport syndrome (2)	Diabetic nephropathy (12)	ATIN (12)	TBM (7)
MN (29)	MCKD (1)	HTN (5)	CNI (1)	Minor glomerular abnormality (12)
ANCA related (21)	Gitelman syndrome (1)			Minimal Change Disease (14)
Lupus nephritis (12)				Not diagnostic (10)
FSGS (11)				Immunotactoid nephropathy (1)
IC-mediated MGPN (6)				Advanced chronic change (1)
Infection-related GN (4)				LCAT deficiency (1)
IgA vasculitis (4)				
C3 nephropathy (1)				
HIV related (1)				

MN, membranous nephropathy; ANCA, anti-neutrophil cytoplasmic antibody; FSGS, focal segmental glomerulonephritis; IC-mediated MPGN, Immune complex mediated membranoproliferative glomerulonephritis; Infection-related GN, Infection-related glomerulonephritis; MCKD, multicystic kidney dysplasia; LCAT, lectin cholesterol acyltransferase; HTN, Hypertensive nephropathy; ATIN, acute tubulointerstitial nephritis; CNI, calcineurin inhibitor toxicity; TBM, thin basement membrane.

Table S3. Univariate and multivariable Cox proportional hazard models for Inflammatory-VMC.

Inflammatory-VMC	Univariate HR [95%CI]	Model 1 HR [95%CI]	Model 2 HR [95%CI]
≥30 cells/mm^2-VMC			
Absent	Reference	Reference	Reference
Present	2.99 (1.71 to 5.33)	2.96 (1.70 to 5.29)	2.21 (1.20 to 4.14)
≥50 cells/mm^2-VMC			
Absent	Reference	Reference	Reference
Present	3.08 (1.77 to 5.46)	3.07 (1.76 to 5.44)	2.20 (1.20 to 4.09)
≥80 cells/mm^2-VMC			
Absent	Reference	Reference	Reference
Present	3.43 (1.97 to 6.01)	3.39 (1.94 to 6.01)	2.63 (1.42 to 5.00)
≥100 cells/mm^2-VMC			
Absent	Reference	Reference	Reference
Present	3.23 (1.86 to 5.70)	3.18 (1.83 to 5.60)	2.42 (1.31 to 4.53)
≥150 cells/mm^2-VMC			
Absent	Reference	Reference	Reference
Present	2.84 (1.63 to 4.96)	2.79 (1.60 to 4.88)	2.10 (1.14 to 3.88)
≥200 cells/mm^2-VMC			
Absent	Reference	Reference	Reference
Present	2.44 (1.40 to 4.24)	2.38 (1.36 to 4.13)	1.94 (1.05 to 3.56

Model 1: Adjusted for age and sex. Model 2: Adjusted for the covariates in model 1, urinary protein excretion and eGFR. VMC, veno-muscular complex; HR, hazard ratio; CI, confidence interval.

Table S4. Univariate and multivariable Cox proportional hazard models for Hyper-VMC.

Hyper-VMC	Univariate HR [95%CI]	Model 1 HR [95%CI]	Model 2 HR [95%CI]
≥550μm VMC-A/W			
Absent	Reference	Reference	Reference
Present	1.14 (0.66 to 1.99)	1.12 (0.64 to 1.97)	1.10 (0.61 to 2.00)
≥850μm VMC-A/W			
Absent	Reference	Reference	Reference
Present	0.76 (0.39 to 1.49)	0.74 (0.38 to 1.45)	0.85 (0.43 to 1.71)
≥1000μm VMC-A/W			
Absent	Reference	Reference	Reference
Present	0.83 (0.35 to 1.94)	0.81 (0.34 to 1.89)	0.81 (0.34 to 1.98)

Model 1: Adjusted for age and sex. Model 2: Adjusted for the covariates in model 1, urinary protein excretion and eGFR. VMC-A/W, area/width of veno-muscular complex; HR, hazard ratio; CI, confidence interval.

Table S5. Clinical and histopathological findings for all patients and for patients stratified by the IFTA score.

IFTA	All patients (n= 246)	IFTA score 0 (n=31)	IFTA score 1 (n= 102)	IFTA score 2 (n=82)	IFTA score3 (n=31)	P for trend
Men, n (%)	131 (53)	13 (43)	50 (49)	49 (60)	19 (61)	0.04*
Age, yr	55.3±16.6	42.0±17.2	56.3±15.8	59.0±14.8	55±16.6	0.003**
BMI, kg/m^2	22.7±3.5	22.4±3.3	23.2±3.5	22.0±3.5	23.4±4.0	0.93
sBP, mmHg	128.5±18.6	121.4±14.1	126.9±18.3	129.5±19.6	138.4±18.5	<0.001**
dBP, mmHg	79.1±13.2	75.5±9.8	78.3±13.0	80.2±14.4	82.7±12.6	0.03*
sCr, mg/dl	1.26±0.84	0.71±0.11	1.06±0.85	1.38±0.71	2.1±0.9	<0.001**
eGFR, ml/min/1.73 m^2	56.0±25.6	85.9±16.2	63.4±23.8	45.5±17.6	29.6±13.5	<0.001**
U-P,[a] g/gCr	1.04 (0.37-3.84)	0.70 (0.19-2.78)	1.01 (0.33-3.10)	1.10 (0.43-4.39)	1.99 (0.49-4.70)	0.04*
Hb A1c, n (%)	5.8±0.7	5.6±0.5	5.8±0.6	5.9±0.9	5.8±0.7	0.19
Hypertension, n (%)	141 (57)	9 (30)	49 (48)	59 (72)	24 (77)	<0.001**
DM, n (%)	53 (22)	2 (7)	19 (19)	23 (28)	9 (29)	0.009**
ACE-I or ARB, n (%)	102 (41)	3 (10)	36 (35)	45 (55)	18 (58)	<0.001**
Ca blockade, n (%)	105 (43)	3 (10)	39 (38)	43 (52)	20 (65)	<0.001**
Statin, n (%)	65 (26)	5 (17)	27 (27)	22 (27)	11 (35)	0.13
Hematuria, n (%)	143 (58)	16 (53)	68 (67)	41 (50)	18 (58)	0.57
VMC A/W, [a] μm	541 (347-814)	496 (284-819)	518 (359-783)	565 (340-811)	665 (445-937)	0.02*
Inflammatory-VMC, n (%)	80 (33)	1 (3)	20 (20)	37 (45)	22 (71)	<0.001**

IFTA, interstitial fibrosis and tubular atrophy; BMI, body mass index; sBP, systolic blood pressure; dBP, diastolic blood pressure; U-P, urinary protein excretion; HbA1c, Hemoglobin A1c; DM, Diabetes mellitus; sCr, serum creatinine; eGFR, estimated glomerular filtration rate; ACE-I, angiotensin–converting enzyme inhibitor; ARB, angiotensin II type 1 receptor blocker; VMC, veno-muscular complex; VMC-A/W, VMC average width. [a] Median (interquartile range). [†]Tests for linear trend across IFTA score. P for trend was calculated by the Cochran-Armitage trend test or the Jonckheere-Terpstra test. * p<0.05, ** p<0.01.

Table S6. Clinical and histopathological findings for all patients and for patients stratified by the Inflammation score.

Inflammation	All patients (n= 246)	Inflammation score 0 (n= 112)	Inflammation score 1 (n= 75)	Inflammation score 2 (n=41)	Inflammation score 3 (n=18)	P for trend
Men, n (%)	131 (53)	62 (55)	34 (45)	25 (61)	10 (56)	0.83
Age, yr	55.3±16.6	54.6±16.8	56.0±16.3	57.0±14.2	52.5±20.2	0.64
BMI, kg/m^2	22.7±3.5	23.0±3.5	22.7±3.4	22.6±3.7	21.2±3.9	0.16
sBP, mmHg	128.5±18.6	124.4±16.4	131.2±19.4	132.2±19.7	133.7±23.0	0.004**
dBP, mmHg	79.1±13.2	76.7±11.1	79.9±13.4	82.5±14.8	83.4±17.2	0.02*
sCr, mg/dl	1.26±0.84	1.06±0.84	1.20±0.70	1.59±0.65	2.0±1.1	<0.001**
eGFR, ml/min/1.73 m^2	56.0±25.6	67.2±25.0	54.3±23.4	38.5±14.9	32.9±13.3	<0.001**
U-P,[a] g/gCr	1.04 (0.37-3.84)	0.83 (0.30-2.86)	1.30 (0.37-3.95)	1.62 (0.52-4.06)	1.28 (0.62-4.84)	0.05*
Hb A1c, n (%)	5.8±0.7	5.9±0.8	5.8±0.6	5.8±0.6	5.4±0.7	0.13
Hypertension, n (%)	141 (57)	55 (49)	45 (60)	32 (78)	9 (50)	0.04*
DM, n (%)	53 (22)	21 (19)	16 (21)	12 (29)	4 (22)	0.28
ACE-I or ARB, n (%)	102 (41)	40 (36)	35 (47)	22 (54)	5 (28)	0.37
Ca blockade, n (%)	105 (43)	37 (33)	36 (48)	24 (59)	8 (44)	0.01*
Statin, n (%)	65 (26)	31 (28)	19 (25)	14 (34)	1 (6)	0.38
Hematuria	143 (58)	63 (56)	47 (63)	25 (61)	8 (44)	0.83
VMC A/W, [a] μm	541 (347-814)	504 (302-748)	560 (346-826)	598 (390-846)	742 (618-1057)	0.02*
Inflammatory-VMC, n (%)	80 (33)	19 (17)	23 (31)	25 (61)	13 (72)	<0.001**

BMI, body mass index; sBP, systolic blood pressure; dBP, diastolic blood pressure; U-P, urinary protein excretion; HbA1c, Hemoglobin A1c; DM, Diabetes mellitus; sCr, serum creatinine; eGFR, estimated glomerular filtration rate; ACE-I, angiotensin–converting enzyme inhibitor; ARB, angiotensin II type 1 receptor blocker; VMC, veno-muscular complex; VMC-A/W, VMC average width. [a] Median (interquartile range). †Tests for linear trend across IFTA score. P for trend was calculated by the Cochran-Armitage trend test or the Jonckheere-Terpstra test. * p<0.05, ** p<0.01.

Table S7. Multi regression analysis of VMC-A/W and clinical variables.

Variables	Coefficient	95% CI	SE	P value
Age [increased by 5yrs]	-3.36	-20.7 to 14.0	8.80	0.70
Gender	-1.56	-51.0 to 47.9	25.1	0.95
eGFR [increased by 10 mL/min/1.73m^2]	-32.9	-53.9 to -11.8	10.68	0.002**
DM	7.23	-54.0 to 68.5	31.1	0.82
Hypertension	17.85	-44.2 to 79.9	31.53	0.57
BMI	10.3	-3.72 to 24.3	7.12	0.15
U-P [increased by 1 g/gCr]	-13.7	-27.0 to -0.48	6.74	0.04*
ACE-I or ARB	-18.1	-76.8 to 40.49	29.7	0.54
Hematuria	8.31	-40.7 to 57.3	24.8	0.73

eGFR, estimated glomerular filtration rate; DM, Diabetes mellitus; BMI, body mass index; U-P, urinary protein excretion; ACE-I, angiotensin–converting enzyme inhibitor; ARB, angiotensin II type 1 receptor blocker; SE; standard error; CI, confidence interval. Model R^2 = 0.063, adjusted R^2 = 0.027. * p<0.05, ** p<0.01.

Table S8. Correlation of VMC-A/W with variables.

	LogVMC-A/W	Age	eGFR	Log-UP
Age	0.0573			
eGFR	-0.23**	-0.3966**		
Log-UP	-0.068	0.182**	-0.07	
BMI	0.0955	0.192**	-0.396**	0.070

Correlation matrix using Spearman correlation coefficients. BMI, body mass index; Log-U-P, log-transformed urinary protein excretion; LogVMC-A/W, log-transformed veno-muscular complex average width. ** $p<0.01$.

Table S9. Univariate and multivariable Cox proportional hazard models for Inflammatory-VMC with or without area correction.

Inflammatory-VMC Without area correction	Univariate HR [95%CI]	Model 1 HR [95%CI]	Model 2 HR [95%CI]	Model 3 HR [95%CI]
≥20 cells/ VMC				
Absent	Reference	Reference	Reference	Reference
Present	3.28 (1.89 to 5.82)	3.28 (1.88 to 5.82)	2.62 (1.44 to 4.89)	1.92 (0.98 to 3.80)
≥50 cells/ VMC				
Absent	Reference	Reference	Reference	Reference
Present	2.91 (1.67 to 5.08)	2.85 (1.63 to 4.99)	2.31 (1.27 to 4.23)	1.84 (0.99 to 3.44)
≥100 cells/ VMC				
Absent	Reference	Reference	Reference	Reference
Present	2.34 (1.32 to 4.08)	2.33 (1.32 to 4.06)	1.96 (1.07 to 3.57)	1.51 (0.80 to 2.80)
Inflammatory-VMC (with area correction)				
Absent	Reference	Reference	Reference	Reference
Present	3.43 (1.97 to 6.01)	3.39 (1.94 to 6.01)	2.69 (1.46 to 5.03)	1.97 (1.003 to 3.91)

Model 1: Adjusted for age and sex. Model 2: Adjusted for urinary protein excretion and eGFR. Model 3: Adjusted for the covariates in model 2 and IFTA. VMC, veno-muscular complex; IFTA, interstitial fibrosis and tubular atrophy; HR, hazard ratio; CI, confidence interval.
